# How perceived organizational support is associated with thriving at work: the chain-mediating roles of professional competence and psychological empowerment among mental health teachers

**DOI:** 10.3389/fpsyg.2026.1820283

**Published:** 2026-08-05

**Authors:** Suizhi Zeng, Zhijun Liu, Chao Wang

**Affiliations:** 1 School of Psychology, Hainan Normal University, Haikou, China; 2 Adolescent Psychological Development and Education Center of Hainan, Hainan Normal University, Haikou, China; 3 Tangxia Junior High School, Dongguan, China

**Keywords:** mental health teachers, perceived organizational support, professional competence, psychological empowerment, thriving at work

## Abstract

**Purpose:**

The research investigated the correlation between perceived organizational support and thriving at work among mental health teachers, emphasizing the mediating effects of professional competence and psychological empowerment.

**Methods:**

A cross-sectional design was used to gather data from 323 mental health teachers via the Perceived Organizational Support Questionnaire, the Professional Competence Questionnaire for Mental Health Teachers, the Psychological Empowerment Scale, and the Thriving at Work Scale. Path analysis and the bootstrap method were applied to examine the relationships and mediating effects among the variables.

**Results:**

The findings demonstrated that perceived organizational support positively predicted thriving at work; additionally, both professional competence and psychological empowerment acted as simple mediators and serially mediated the relationship between perceived organizational support and thriving at work.

**Discussion:**

The study elucidates the internal mechanism by which perceived organizational support is associated with thriving at work, thereby filling a research gap related to mental health teachers, establishing a theoretical and empirical basis for future investigations, and providing actionable recommendations for school administrators to support thriving at work among mental health teachers.

## Introduction

Thriving at work is defined as a psychological state in which individuals experience both vitality and learning in their professional environment (Spreitzer et al., 2005). Kleine et al. (2023) conducted a longitudinal study that revealed a substantial positive correlation between workplace thriving and overall health. A meta-analysis indicated that thriving at work is associated with individual health, attitudes, and performance (Kleine et al., 2019). Thus, thriving at work is important for individual development. The education sector, characterized by its rewarding yet challenging nature, has attracted increasing research attention to the concept (Wang et al., 2021; Wang and Liu, 2019). Educators are increasingly required to continuously develop their expertise (Nazaretsky et al., 2022), adjust to continual curriculum modifications (Jiang, 2022; Xu and Fan, 2017), and undertake various responsibilities outside traditional teaching (Yang et al., 2019). In light of swift societal advancement and ongoing innovations in educational methodologies, educators must adapt to changing pedagogical strategies (Kasneci et al., 2023), which place greater demands on their ability to thrive at work. However, mental health teachers, as a distinct subgroup of teachers, face greater challenges. China’s exam-centric education system prioritizes academic performance, predominantly measured by standardized test results, as the principal criterion for assessing students and educational institutions (Gao, 2006). In this context, mental health education, as a non-essential subject, frequently holds a peripheral status within the school curriculum and lacks adequate institutional focus. As a result, educators in the sector are often deprived of professional development opportunities, instructional materials, and career advancement. Simultaneously, they are frequently tasked with many non-teaching and miscellaneous duties, which significantly detract from their ability to foster students’ psychological well-being (Wang, 2025). While the question of how educators can thrive at work has attracted considerable research attention, within the Chinese cultural context, most existing studies on thriving at work have focused primarily on the general education sector, paying scant attention to mental health teachers. Among the available studies, a large body of research has found that perceived organizational support positively predicts thriving at work (Paterson et al., 2014; Abid et al., 2015). Other studies have found that perceived organizational support has a positive effect on improving individuals’ competence (Taylor and Alfred, 2010). Interestingly, some studies have also found that individuals who perceive organizational support gain greater self-efficacy, autonomy, and a sense of meaning—that is, a higher level of psychological empowerment (Musenze et al., 2021). These studies suggest that organizational support, as an external contextual variable, may contribute to higher levels of two internal variables—competence and psychological empowerment. This process can be viewed as a form of social exchange, wherein individuals reciprocate organizational support with increased competence and a heightened sense of empowerment (Cropanzano and Mitchell, 2005). Other researchers have indicated that these internal variables may manifest as a good psychological state, specifically thriving at work (Wang et al., 2020; Xu L. et al., 2025), and the Integrated Model of Human Growth at Work provides theoretical support for this inference (Spreitzer and Porath, 2014). This suggests that competence and psychological empowerment may emerge as important mediating variables in the process through which perceived organizational support translates into thriving at work. More importantly, drawing on Self-determination Theory, researchers have also found that an individual’s competence is beneficial to psychological needs such as autonomy and self-efficacy, which are essential components of psychological empowerment (Deci and Ryan, 2000; Huang et al., 2025). However, prior studies have separately examined the mediating roles of competence (Wang and Liu, 2018) and psychological empowerment (Guan and Frenkel, 2021) in the relationship between perceived organizational support and thriving at work, yet they have not integrated both into a single model, let alone tested the possible chain mediation mechanism between perceived organizational support and thriving at work. Particularly for the group of mental health teachers, examining this serial mediation pathway is especially important, as it helps clarify how organizational support sequentially contributes to thriving at work through the stepwise transmission of competence and psychological empowerment, thereby filling the gap in existing research. Therefore, drawing on Social Exchange Theory, the Integrated Model of Human Growth at Work and Self-determination Theory, this study aims to explore the mediating mechanism of the relationship between perceived organizational support and thriving at work among mental health teachers, and from the perspective of individual psychological resources, focuses on examining professional competence and psychological empowerment as two potential mediating variables.

### Perceived organizational support and thriving at work

Perceived organizational support refers to employees’ general beliefs regarding the degree to which the organization appreciates their contributions and prioritizes their well-being (Eisenberger et al., 1986). Within the framework of traditional Chinese culture, Chen (2006) identified two levels of perceived organizational support: emotional and instrumental support. Social Exchange Theory indicates that one party’s behavior is contingent upon the other party’s behavior. This process begins when at least one party makes an “action”; if the other party reciprocates, a new round of exchange is initiated (Cropanzano and Mitchell, 2005). Empirical studies have indicated that when employees receive support to engage in tasks that leverage their strengths, they are more likely to feel capable of effectively meeting job demands, which stimulates individual growth and development as they have the opportunity to further cultivate their core skills and abilities (Guan and Frenkel, 2021). A prior study has suggested that perceived organizational support positively predicts thriving at work (Xu H. et al., 2025). Meng et al. (2024) also discovered that organizational support fosters employees’ vitality and learning, and that these states are conducive to innovative behavior. In conclusion, perceived organizational support is probably associated with thriving at work.

### The mediating role of professional competence

Competence is an intrinsic trait of an individual that correlates with criterion-referenced exemplary work performance (Spencer and Spencer, 1993). Competencies encompass conventional cognitive abilities such as reading, writing, and mathematics, alongside historically acknowledged personality traits, including communication skills, patience, moderate goal setting, and ego development (McClelland, 1973). The Competency Iceberg Model acknowledges that knowledge and skill competencies are typically apparent and superficial, whereas self-concept and trait competencies are more concealed and profound (Spencer and Spencer, 1993). The professional competence of mental health teachers refers to personal attributes such as knowledge, concepts, abilities, and personality traits that are related to job positions, organizational environment, cultural atmosphere, and work performance (Wang and Zhang, 2011). The competencies of these educators are likely to contribute to their professional success. A previous study demonstrated an association between emotional competence and teacher burnout, indicating that educators with reduced emotional competence are at an increased risk of burnout and may struggle to access available external and internal resources (Fiorilli et al., 2017). Other studies have shown that teachers’ social–emotional competency contributes to their workplace thriving (Collie and Perry, 2019). Notably, there is an intrinsic link between perceived organizational support and competency. According to Social Exchange Theory, individuals respond to the benefits they receive with goodwill or help toward the party with whom they have a social exchange relationship (Cropanzano and Mitchell, 2005). Therefore, organizational support such as training, compensation, incentives, and mentoring makes employees feel valued and motivates them to enhance their job competencies to achieve organizational goals (Sitindaon et al., 2021). Empirical research has indicated that perceived organizational support can contribute to teachers’ family-school partnership competency, that is, their ability to develop and maintain constructive home-school relationships (Wei and Zhang, 2025). Other researchers have also found a significant positive correlation between perceived organizational support and competency (Poikkeus et al., 2020). Wang and Liu (2018) pointed out that school support can provide teachers with a teaching and working environment that conveys a tangible sense of support; when teachers feel the school’s care and support, they will put more effort into working for the students and the school. This, in turn, helps teachers improve their teaching competency and professional pride, while also facilitating the establishment of positive interpersonal relationships and social support systems. In summary, professional competence is likely to serve as a mediator between perceived organizational support and thriving at work.

### The mediating role of psychological empowerment

Psychological empowerment is characterized by heightened intrinsic task motivation, comprising four cognitive elements: sense of impact, self-efficacy, meaningfulness, and choice. Impact denotes the extent to which an individual’s actions are regarded as “making a difference” in achieving the task’s objectives, namely generating desired outcomes within the task context. Meaningfulness pertains to the significance of the task’s objective or purpose, evaluated against the individual’s personal ideas or standards, which indicates the individual’s inherent concern for a specific task. Self-efficacy pertains to an individual’s confidence in their capability to perform work-related tasks proficiently. Choice encompasses the causal accountability for one’s actions (Thomas and Velthouse, 1990). The Integrated Model of Human Growth at Work asserts that three essential components—autonomy, self-efficacy, and relatedness—facilitate thriving at work (Spreitzer and Porath, 2014). An empirical study indicates that when individuals believe that they can creatively complete work even in difficult situations, they are more likely to feel alert, active, and energetic in their learning, thereby enhancing their sense of thriving at work (Christensen-Salem et al., 2021). Ochoa Pacheco and Coello-Montecel (2023) discovered that individuals who perceive themselves as empowered to manage their work and recognize its significance are more inclined to have heightened energy and motivation in the workplace. Zhuang et al. (2025) assert that individuals with elevated psychological empowerment engage actively in formulating and modifying care plans, and this self-determination subsequently fosters nurses’ professional thriving at work. Caesens and Stinglhamber (2014) found that the more employees perceive organizational support and value, the higher their self-efficacy. Other research has found that employees who receive organizational support perceive their work as more meaningful (Akgunduz et al., 2018). Research has also indicated that organizational support—not only material support, but also emotional support, fair treatment, the provision of development opportunities, and participation in decision-making—is a key prerequisite for stimulating work autonomy (Saad and Elsayed, 2019). Other researchers indicate that perceived organizational support correlates positively with psychological empowerment, implying that when an organization attends to its employees, the workers experience an enhanced sense of responsibility for their activities (Meira and Hancer, 2021). In conclusion, psychological empowerment is likely to serve as a mediator between perceived organizational support and thriving at work.

### The chain mediating role of professional competence and psychological empowerment

According to self-determination theory, every individual has basic needs for competence and autonomy (Deci and Ryan, 2000), and these two needs are the core elements of psychological empowerment (Thomas and Velthouse, 1990). Maior et al. (2020) indicated that individuals with higher competence are more capable of creating or leveraging their environment to satisfy their own needs for a sense of autonomy, competence (self-efficacy), and relatedness, thereby experiencing less emotional exhaustion and a higher level of personal accomplishment. This suggests that competence may be positively associated with psychological empowerment. The findings of Huang et al. (2025) are consistent with this speculation, demonstrating that the higher the teachers’ competence is, the higher their psychological empowerment is. Other research has also found that high competence enables employees to experience a greater sense of work value, feel a stronger impact within the organization, and further develop their self-efficacy to achieve organizational goals, all of which ultimately lead to higher work performance (Ochoa Pacheco and Coello-Montecel, 2023). Similarly, we speculate that for mental health teachers, a higher level of professional competence allows them to perform teaching and counseling more effectively and to apply their expertise proactively. This, in turn, contributes to their self-confidence and initiative, making them feel that their work is meaningful and has a positive impact on others. In summary, professional competence and psychological empowerment together may function as serial mediators in the relationship between perceived organizational support and thriving at work.

### The present study

**Figure 1:**
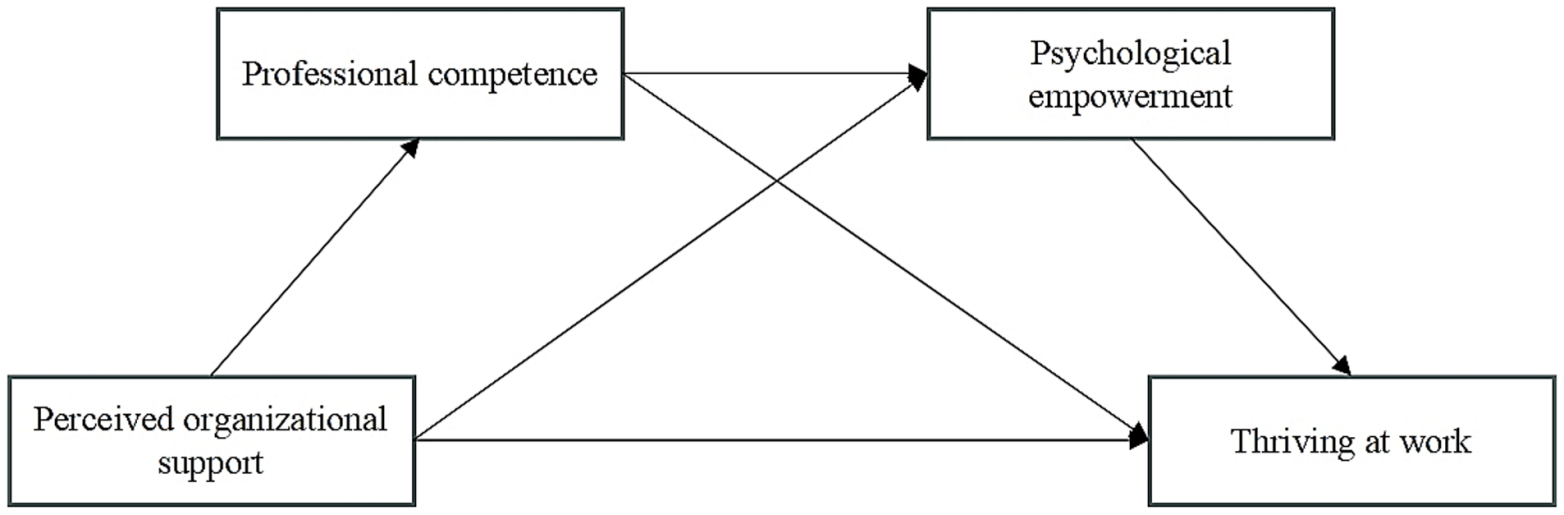
The conceptual framework of the study.

As shown in Figure 1, the hypothesized model is used to investigate the underlying mechanisms through which perceived organizational support is linked to thriving at work. The hypotheses were as follows:

Hypothesis 1: Perceived organizational support positively predicts thriving at work.

Hypothesis 2: Professional competence plays a mediating role between perceived organizational support and thriving at work.

Hypothesis 3: Psychological empowerment plays a mediating role between perceived organizational support and thriving at work.

Hypothesis 4: Professional competence and psychological empowerment serially mediate the relationship between perceived organizational support and thriving at work.

## Methods

### Participants and procedure

Before the study, an *a priori* power analysis was conducted with a significance level of *α* = 0.05, power (1 − β) = 0.80, an effect size of 0.15, and 3 predictors. According to G*Power, the minimum required sample size was 77 (Faul et al., 2009). The participants in this study were 323, a sample size far exceeding the minimum requirement.

Data for this study were collected through a combination of offline and online questionnaires. The offline survey employed convenience sampling: first, several primary and secondary schools in Haikou City were selected, and questionnaires were then distributed to the mental health teachers at these schools. The online survey also employed convenience sampling: after being admitted to several QQ groups for mental health teachers (QQ, a widely used instant messaging platform in China) and obtaining the group administrators’ permission, we shared a QR code, generated by Wenjuanxing (an online survey platform) and linking to the questionnaire, and invited the teachers to scan the code and complete the questionnaire. A total of 351 questionnaires were distributed in this study. After excluding questionnaires with a response time of less than 180 s, patterned responses, or contradictory answers, 323 valid questionnaires were obtained, yielding an effective response rate of 92%. The specific composition of the sample is shown in Table 1.

**Table 1:** Distribution table of participant conditions.

Demographic characteristics	Categories	Count	Proportion
Gender	Male	90	27.9
	Female	233	72.1
Age	20–30 years old	224	69.3
	31–40 years old	73	22.6
	41–50 years old	17	5.3
	Over 51 years old	9	2.8
Qualification	Associate degree	20	6.2
	Bachelor’s degree	210	65
	Master’s Degree	93	28.8
School category	Primary school	102	31.6
	Junior high school	99	30.7
	High school	92	28.5
	Secondary vocational school	30	9.3
School location	Village	44	13.6
	County	122	37.8
	City	157	48.6
Work schedules	Full-time	167	51.7
	Part-time	156	48.3
Job tenure	Under 3 years	204	63.2
	3–5 years	56	17.3
	6–10 years	35	10.8
	10 years and above	28	8.7
Title	Uncertified	175	54.2
	Third-level teacher	7	2.2
	Second-level teacher	78	24.1
	First-level teacher	50	15.5
	Senior teacher	11	3.4
	Principal teacher	2	0.6
First specialty	Psychology	133	41.2
	Education	90	27.9
	Other	100	31.0
Outstanding teacher	Yes	58	18.0
	No	265	82.0

The study was approved by the Ethics Committee of the School of Psychology and conducted in accordance with institutional guidelines and the Declaration of Helsinki. All participants willingly submitted written informed consent after being informed of the relevant details of the research. The collected questionnaires and consent forms were stored separately to ensure that participants could not be identified. Before the administration, the experimenter informed the participants that they had the right to decide whether to participate or not and that they could withdraw at any time, and explicitly stated that all personal information would be kept strictly confidential.

### Measurements

#### Perceived organizational support

The Perceived Organizational Support Questionnaire, developed by Chen (2006), was used. It consists of two dimensions: emotional support (e.g., “The organization values my contributions.”) and instrumental support (e.g., “The organization will make every effort to provide me with a favorable working environment and necessary facilities.”), totaling 14 items. A 7-point Likert scale was employed, with values spanning from 1 (strongly disagree) to 7 (strongly agree), and the ratings for each dimension were aggregated to derive a total score for perceived organizational support. An elevated score on this scale signifies an increased perception of organizational support. The Cronbach’s alpha coefficient for this scale in the present investigation was 0.97. A confirmatory factor analysis was performed to assess the structural validity of the questionnaire. The findings demonstrated a favorable model fit: χ^2^/*df* = 2.26, CFI = 0.98, TLI = 0.98, RMSEA = 0.07.

#### Professional competence questionnaire for mental health teachers

The Professional Competence Questionnaire was developed by Zhuang (2019). The questionnaire comprises four dimensions: teaching and consulting ability (12 items; e.g., “In psychology classes, timely and targeted guidance and feedback can be provided to students.”), research and mentorship skills (9 items; e.g., “Capable of swiftly and accurately capturing information pertinent to counseling.”), professional development skills (7 items; e.g., “Understand students’ interests and hobbies, and gain substantial insights from them.”), and professional charisma (9 items; e.g., “Adept at persuading, influencing, or motivating students through one’s verbal and nonverbal communication.”), encompassing 37 items in total. A 6-point Likert scale was employed, with ratings ranging from 1 (strongly disagree) to 6 (strongly agree), and the scores of all 37 items were summed to produce a total professional competence score. A higher score on this measure indicates a greater level of professional competence among mental health teachers. The Cronbach’s alpha coefficient for this scale in the present study was 0.98. A confirmatory factor analysis was performed to assess the structural validity of the questionnaire. The findings demonstrated a satisfactory model fit: χ^2^/*df* = 2.13, CFI = 0.98, TLI = 0.97, RMSEA = 0.06.

#### Psychological empowerment

The Psychological Empowerment Scale, developed by Spreitzer (1995), was utilized. The questionnaire comprises four dimensions: meaning (“My job activities are personally meaningful to me”), self-efficacy (“I am confident about my ability to do my job”), self-determination (“I have significant autonomy in determining how I do my job”), and impact (“My impact on what happens in my department is substantial”), totaling 12 items. A 5-point Likert scale was employed, with ratings ranging from 1 (strongly disagree) to 5 (strongly agree), and the scores of all 12 items were summed to produce a total psychological empowerment score. An elevated score on this measure signifies an increased degree of psychological empowerment. The Cronbach’s alpha coefficient for this scale in the present study was 0.92. Confirmatory factor analysis was performed to assess the structural validity of the questionnaire. The findings demonstrated a favorable model fit: χ^2^/*df* = 3.06, CFI = 0.97, TLI = 0.96, RMSEA = 0.08.

#### Thriving at work

The Thriving at Work Scale, developed by Porath et al. (2012), was used. The scale comprises two dimensions: meaning (“I find myself learning often”) and vitality (“I feel alive and vital”), totaling 10 items. A 5-point Likert scale was employed, with values ranging from 1 (strongly disagree) to 5 (strongly agree), and the scores of all 10 items were summed to produce a total thriving at work score. An elevated score on this measure signifies a higher level of thriving at work. The Cronbach’s alpha coefficient for this scale in the present study was 0.95. Confirmatory factor analysis was performed to assess the structural validity of the questionnaire. The results demonstrated a strong model fit: χ^2^/*df* = 2.63, CFI = 0.98, TLI = 0.97, RMSEA = 0.07.

### Data analysis

We used R 4.5.1 with R Studio/2024.04.2+764 as an integrated development environment (IDE) to analyze the data. The R package psych was utilized to conduct reliability analysis, evaluate common method bias, and calculate descriptive statistics (Revelle, 2026). The R package lavaan, which can apply the maximum likelihood method for model estimation and evaluation, was employed to execute confirmatory factor analysis (CFA) and assess the serial mediation effects within Path Analysis when using bootstrap resampling (Rosseel et al., 2025).

## Results

### Common method bias test

Tang and Wen (2020) advised performing a common method bias check using the unmeasured latent method construct (ULMC). Initially, a baseline model (M1) with only the substantive trait factors was developed. Secondly, a bifactor model (M2) incorporating both trait factors and a common method factor was constructed. The changes in model fit indices between M1 and M2 were as follows: ΔCFI = 0.043, ΔTLI = 0.038, ΔRMSEA = 0.006. The inclusion of the method factor led to a rise in CFI and TLI of less than 0.1, and a fall in RMSEA of less than 0.05, indicating that the addition of the method factor did not yield a substantial enhancement in model fit, thus suggesting that the common method bias in the research is not serious.

### Correlation analysis of the study variables

Table 2 presents the correlations among the variables in the study. The findings indicated that perceived organizational support was positively correlated with professional competence, psychological empowerment, and thriving at work. Professional competence was positively connected with psychological empowerment and thriving at work. Psychological empowerment was positively connected with thriving at work.

**Table 2:** Means, standard deviations, and correlations between variables

Variables	M	SD	1	2	3	4	5	6
1.Gender	—	—	1.00					
2.Age	—	—	0.14*	1.00				
3.PS	4.64	1.29	-0.23***	-0.12*	1.00			
4.PC	4.70	0.73	0.01	0.20***	0.40***	1.00		
5.PE	3.68	0.65	-0.05	0.20***	0.47***	0.69***	1.00	
6.TW	3.91	0.67	-0.04	0.12*	0.47***	0.66***	0.74***	1.00

Note: POS: Perceived organizational support; PC: Professional competence; PE: Psychological empowerment; TW: Thriving at work; In gender and age coding: Male = 1, Female = 0. Age: 20–30 = 1, 31–40 = 2, 41–50 = 3, Over 51 = 4.

### Analysis of mediated effects

Following correlation analysis, we subsequently conducted path analysis to investigate the mechanism by which perceived organizational support contributes to thriving at work. The model was estimated with the Maximum Likelihood method. Gender and age were controlled as covariates to facilitate a precise analysis of the path coefficients. The findings presented in Table 3 demonstrated that perceived organizational support significantly and positively predicted thriving at work (*β* = 0.14, *p* < 0.05). It also significantly predicted the professional competence (*β* = 0.44, *p* < 0.001) and psychological empowerment (*β* = 0.31, *p* < 0.001). The professional competence was a significant positive predictor of both psychological empowerment (*β* = 0.53, *p* < 0.001) and thriving at work (*β* = 0.26, *p* < 0.001). Ultimately, psychological empowerment was a strong predictor of thriving at work (*β* = 0.50, *p* < 0.001).

**Table 3:** Direct effects in the path analysis results.

Independent variables	Outcome variables	*β*	*z*	*p*
Age	Professional competence	0.24	4.79	<0.001
Gender		0.08	1.49	>0.05
Perceived organizational support		0.44	8.63	<0.001
Age	Psychological empowerment	0.13	3.35	<0.001
Gender		−0.01	−0.13	>0.05
Perceived organizational support		0.31	7.15	<0.001
Professional competence		0.53	12.36	<0.001
Age	Thriving at work	−0.03	−0.71	>0.05
Gender		0.02	0.48	>0.05
Perceived organizational support		0.14	3.17	<0.01
Professional competence		0.26	5.29	<0.001
Psychological empowerment		0.50	9.37	<0.001

**Figure 2:**
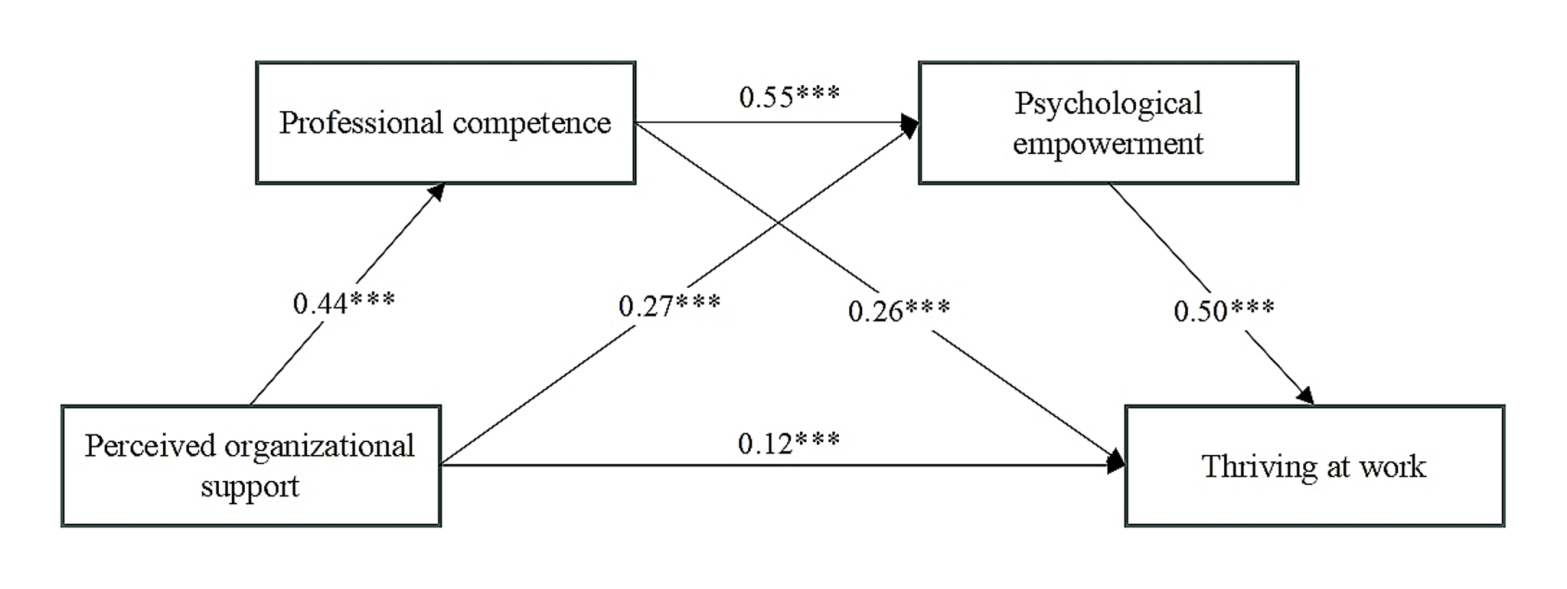
Chain mediation model.

Subsequently, mediation analysis was conducted. The proposed mediation model presented in Figure 2 encompasses three indirect pathways: indirect effect 1: perceived organizational support → professional competence → thriving at work; indirect effect 2: perceived organizational support → psychological empowerment → thriving at work; indirect effect 3: perceived organizational support → professional competence → psychological empowerment → thriving at work. The significance of these indirect effects was evaluated using the bias-corrected percentile bootstrap method with 5,000 resamples in R. A mediating effect is deemed statistically significant if its 95% bias-corrected bootstrap confidence interval excludes zero. The results presented in Table 4 indicated that indirect effect 1 (95% confidence interval [0.02, 0.11]), indirect effect 2 (95% confidence interval [0.03, 0.10]), and indirect effect 3 (95% confidence interval [0.04, 0.09]) all excluded zero, thus confirming the significance of each indirect pathway.

**Table 4:** Indirect effects in the path analysis results.

Path	*β*	95% CI		Path effect proportion
		Lower	Upper	
POS → TW	0.14***	0.03	0.12	0.26
POS → PC → TW	0.12***	0.03	0.09	0.23
POS → PE → TW	0.15***	0.05	0.11	0.28
POS → PC → PE → TW	0.12***	0.04	0.08	0.23

POS, perceived organizational support, PC, professional competence, PE, psychological empowerment, TW, thriving at work.

## Discussion

This study investigated the correlation between perceived organizational support and thriving at work, while assessing the roles of professional competence and psychological empowerment as serial mediators in this relationship. The study’s findings are analyzed in conjunction with the current literature. The study’s shortcomings and implications are subsequently examined, accompanied by recommendations for future research.

### The direct effect of perceived organizational support

The findings indicated that perceived organizational support predicted thriving at work, supporting Hypothesis 1. Consistent with Social Exchange Theory (Cropanzano and Mitchell, 2005), organizations supply financial, physical, and psychological resources, as well as task-related, interpersonal, and developmental opportunities required by employees; in return, employees reciprocate with a state of vigor (Guan and Frenkel, 2021). Notably, this study recruited mental health teachers as participants and obtained results consistent with previous studies (Li and Liu, 2023; Meng et al., 2024), thereby extending the applicability of this conclusion to this specific sample and laying an empirical foundation for future related research.

### The simple mediating mechanism analysis

Results indicated that professional competence served as a mediator between perceived organizational support and thriving at work, supporting Hypothesis 2. This outcome supports previous studies (Collie and Perry, 2019; Wei and Zhang, 2025). When mental health teachers perceive organizational support in their work, they have a greater propensity for self-improvement, higher professional competence, and stronger research capability (Sitindaon et al., 2021). Individuals with elevated competence demonstrate a heightened eagerness to acquire new knowledge and skills, thereby displaying higher thriving at work.

In addition, the results also supported Hypothesis 3. Psychological empowerment includes four cognitive elements: sense of impact, self-efficacy, meaningfulness, and autonomy (Thomas and Velthouse, 1990). When mental health teachers perceive their efforts as acknowledged and rewarded, they dedicate increased energy and time to their responsibilities and cultivate greater confidence in their capacity to address diverse issues (Yu et al., 2024), and they have greater job autonomy (Gillet et al., 2013) and experience more work meaningfulness (Akgunduz et al., 2018). When individuals act out of enjoyment or interest (rather than for instrumental reasons), they invest effort more willingly, thereby enhancing their sense of vitality (Mahomed and Rothmann, 2020). Moreover, when individuals believe that they can complete work creatively even in difficult situations, they are more likely to feel alert, alive, and energetic in their learning, thus improving their thriving at work (Christensen-Salem et al., 2021). Finally, positive meaning helps individuals reappraise events as opportunities for growth rather than potential losses, thereby promoting employees’ experience of thriving at work (Guan and Frenkel, 2021).

### The serial mediating mechanism analysis

The present research indicated that professional competence and psychological empowerment served as serial mediators between perceived organizational support and thriving at work, supporting Hypothesis 4. Among these findings, professional competence positively predicted psychological empowerment, which aligns with prior studies (Maior et al., 2020). Competence encompasses conventional cognitive abilities, including communication skills, patience, moderate goal setting, and self-development (McClelland, 1973). Mental health teachers with high professional competence are more adept at sharing work-related ideas and emotions, which is linked to a stronger sense of work meaningfulness; moreover, high professional competence is associated with their competitive advantage in the workplace, which in turn contributes to their self-efficacy, plays a role in organizational decision-making and practice, and thus strengthens their perception of impact on their work (Ochoa Pacheco and Coello-Montecel, 2023). Psychological empowerment manifests as agency, proactivity, and goal-directedness at work, which is conducive to thriving among mental health teachers. For instance, higher self-efficacy and autonomy contribute to vitality, while engaging in meaningful and impactful work facilitates learning (Kaur and Ren, 2024). In conclusion, organizational support contributes to professional competence. Greater competence, in turn, strengthens teachers’ psychological empowerment, fostering thriving at work characterized by vitality and learning.

### Limitations and future directions

First, the measurement of perceived organizational support has certain limitations; specifically, it is important to acknowledge that organizational support was assessed solely through two dimensions: emotional and instrumental support. To address this limitation, future researchers may integrate supervisor and co-worker support into the assessment of perceived organizational support, facilitating a more thorough examination of its correlation with thriving at work. Subsequent research could investigate how the four kinds of organizational support — emotional, instrumental, supervisor, and co-worker support — differentially impact the two dimensions of thriving at work: learning and vitality. Second, because all measurements in this study were obtained from the same respondents (self-report bias) and the questionnaires were administered under the same measurement conditions (same time and place), common method bias is inevitable. Future research could measure predictor and outcome variables from different sources, at different times and places, in order to minimize the variance caused by common method that is unrelated to the study. Third, the cross-sectional methodology restricts the examination of temporal changes or the delayed impact of predictors on outcomes, thus compromising the accuracy of the findings. Consequently, future researchers might employ a longitudinal design to more effectively investigate the mechanisms by which organizational support contributes to thriving at work. The study obtained its sample through convenience sampling via both online and offline channels. As a non-probability sampling method, it is prone to sampling bias, which limits the generalizability of the findings. Future research should adopt probability sampling, using a national sampling frame of primary and secondary schools, randomly selecting schools, and then administering the survey to the psychology teachers in the selected schools. This would effectively enhance the representativeness of the sample and improve the external validity of the conclusions. Finally, at the cultural level, Chinese society as a whole exhibits cultural tendencies of high power distance and collectivism. Mental health teachers growing up in this environment, compared with individuals from low power distance and individualistic cultures, may be more sensitive to organizational support and more willing to reciprocate it (Stinglhamber and Caesens, 2021). This means that the conclusions of this study may be limited in terms of cross-cultural generalizability. Future research could conduct cross-cultural comparisons across countries with different cultural contexts to examine whether the relationships among variables change. Moreover, at the individual level, individuals vary in their power distance and collectivism/individualism tendencies. Existing research has pointed out that when these two cultural tendencies are incorporated as moderating variables into the model, the direction of their moderating effects is inconsistent—they may either strengthen or weaken the relationship (Stinglhamber and Caesens, 2021). However, this study did not measure these individual cultural tendencies, and thus could not test their moderating effects. Building on the current model, future research could further investigate the moderating roles of power distance and collectivism/individualism in order to clarify the boundary conditions.

### Practical implications

This study offers valuable insights for the management practices of mental health teachers, and can support the design of intervention programs and policy-making in the following three aspects, thereby enhancing their thriving at work. First, the findings indicate that perceived organizational support contributes to thriving at work. This suggests that school administrators should guarantee the authorized staffing positions of full-time mental health teachers, ensure they receive professional treatment equivalent to that of head teachers, clearly define the boundaries of their job responsibilities, reduce the imposition of administrative chores unrelated to their professional duties, proactively provide supporting resources for their mental health education activities, and offer them due care and assistance, thereby contributing to an increase in their thriving at work. Second, this study reveals the mediating role of psychological empowerment between perceived organizational support and thriving at work. This indicates that school administrators can enhance mental health teachers’ sense of professional meaning and perceived impact through approaches such as public recognition and sharing of outstanding achievements. At the same time, administrators should reduce administrative intervention and grant mental health teachers sufficient job autonomy, thereby enabling them to maintain vitality and a state of active learning in their mental health education work. Third, this study finds that perceived organizational support can contribute to mental health teachers’ thriving at work through professional competence. It is worth noting that this competence in mental health work can further play a role in thriving at work through psychological empowerment. This suggests that school administrators should attach importance to training in professional knowledge and skills for mental health teachers, establish a regular professional supervision mechanism, and encourage them to conduct psychological research grounded in practice, thus enhancing their professional competence and ultimately enabling them to thrive at work.

## Conclusion

The research indicated that perceived organizational support was positively associated with thriving at work, professional competence, and psychological empowerment. Professional competence and psychological empowerment each served as a mediator, and together they exerted a serial indirect effect. It should be noted that although these direct and indirect associations are statistically significant, the study design does not support causal inferences or conclusions about temporal precedence.

## Data Availability

The raw data supporting the conclusions of this article will be made available by the authors, without undue reservation.

## References

[ref1] Abid G. Zahra I. Ahmed A. (2015). Mediated mechanism of thriving at work between perceived organization support, innovative work behavior and turnover intention. Pak. J. Commer. Soc. Sci. 9, 982–998.

[ref2] Akgunduz Y. Alkan C. Gök Ö. A. (2018). Perceived organizational support, employee creativity and proactive personality: the mediating effect of meaning of work. J. Hosp. Tour. Manag. 34, 105–114. doi: 10.1016/j.jhtm.2018.01.004

[ref3] Caesens G. Stinglhamber F. (2014). The relationship between perceived organizational support and work engagement: the role of self-efficacy and its outcomes. Eur. Rev. Appl. Psychol. 64, 259–267. doi: 10.1016/j.erap.2014.08.002

[ref4] Chen Z. (2006). Chinese Knowledge-Workers’ Perceived Organizational Support and its Influence on their Job Performance and Turnover Intention. Wuhan: Huazhong University of Science & Technology.

[ref5] Christensen-Salem A. Walumbwa F. O. Hsu C. I.-C. Misati E. Babalola M. T. Kim K. (2021). Unmasking the creative self-efficacy–creative performance relationship: the roles of thriving at work, perceived work significance, and task interdependence. Int. J. Hum. Resour. Manag. 32, 4820–4846. doi: 10.1080/09585192.2019.1710721, 37339054

[ref6] Collie R. J. Perry N. E. (2019). Cultivating teacher thriving through social-emotional competence and its development. Aust. Educ. Res. 46, 699–714. doi: 10.1007/s13384-019-00342-2

[ref7] Cropanzano R. Mitchell M. S. (2005). Social exchange theory: an interdisciplinary review. J. Manag. 31, 874–900. doi: 10.1177/0149206305279602, 41677914

[ref8] Deci E. L. Ryan R. M. (2000). The "what" and "why" of goal pursuits: human needs and the self-determination of behavior. Psychol. Inq. 11, 227–268. doi: 10.1207/S15327965PLI1104_01

[ref9] Eisenberger R. Huntington R. Hutchison S. Sowa D. (1986). Perceived organizational support. J. Appl. Psychol. 71, 500–507. doi: 10.1037/0021-9010.71.3.500

[ref10] Faul F. Erdfelder E. Buchner A. Lang A.-G. (2009). Statistical power analyses using G*power 3.1: tests for correlation and regression analyses. Behav. Res. Methods 41, 1149–1160. doi: 10.3758/BRM.41.4.1149, 19897823

[ref11] Fiorilli C. Albanese O. Gabola P. Pepe A. (2017). Teachers' emotional competence and social support: assessing the mediating role of teacher burnout. Scand. J. Educ. Res. 61, 127–138. doi: 10.1080/00313831.2015.1119722

[ref12] Gao L. (2006). Reconstruction of the academic evaluation system for senior high school students. J. South China Normal Univ. Soc. Sci. Ed., 98–102.

[ref13] Gillet N. Gagné M. Sauvagère S. Fouquereau E. (2013). The role of supervisor autonomy support, organizational support, and autonomous and controlled motivation in predicting employees' satisfaction and turnover intentions. Eur. J. Work Organ. Psychol. 22, 450–460. doi: 10.1080/1359432X.2012.665228, 37339054

[ref14] Guan X. Frenkel S. (2021). Organizational support and employee thriving at work: exploring the underlying mechanisms. Pers. Rev. 50, 935–953. doi: 10.1108/PR-10-2019-0569

[ref15] Huang S. Gan L. Ping A. N. (2025). The relationship between social-emotional competence and burnout of kindergarten teacher: the chain mediating role of psychological empowerment and positive coping styles. Stud. Psychol. Behav. 23, 847–853.

[ref16] Jiang A. L. (2022). Identity work as ethical self-formation: the case of two Chinese English-as-foreign-language teachers in the context of curriculum reform. Front. Psychol. 12:774759. doi: 10.3389/fpsyg.2021.774759, 35082721 PMC8784658

[ref17] Kasneci E. Sessler K. Kuechemann S. Bannert M. Dementieva D. Fischer F. . (2023). ChatGPT for good? On opportunities and challenges of large language models for education. Learn. Individ. Differ. 103:102274. doi: 10.1016/j.lindif.2023.102274

[ref18] Kaur M. Ren H. (2024). Navigating dissimilarity: how motivational cultural intelligence enhances psychological empowerment and thriving at work. Group Organ. Manag. 51, 1812–1842. doi: 10.1177/10596011241300635

[ref19] Kleine A.-K. Rudolph C. W. Schmitt A. Zacher H. (2023). Thriving at work: an investigation of the independent and joint effects of vitality and learning on employee health. Eur. J. Work Organ. Psychol. 32, 95–106. doi: 10.1080/1359432X.2022.2102485

[ref20] Kleine A.-K. Rudolph C. W. Zacher H. (2019). Thriving at work: a meta-analysis. J. Organ. Behav. 40, 973–999. doi: 10.1002/job.2375

[ref21] Li Q. Liu M. (2023). The effect of family supportive supervisor behavior on teachers’ innovative behavior and thriving at work: a moderated mediation model. Front. Psychol. 14:1129486. doi: 10.3389/fpsyg.2023.1129486, 36968709 PMC10030523

[ref22] Mahomed F. E. Rothmann S. (2020). Strength use, training and development, thriving, and intention to leave: the mediating effects of psychological need satisfaction. S. Afr. J. Psychol. 50, 24–38. doi: 10.1177/0081246319849030

[ref23] Maior E. Dobrean A. Păsărelu C.-R. (2020). Teacher rationality, social-emotional competencies and basic needs satisfaction: direct and indirect effects on teacher burnout. J. Evid.-Based Psychother. 20, 135–152. doi: 10.24193/jebp.2020.1.8

[ref24] McClelland D. C. (1973). Testing for competence rather than for "intelligence". Am. Psychol. 28, 1–14. doi: 10.1037/h0034092, 4684069

[ref25] Meira J. V. D. S. Hancer M. (2021). Using the social exchange theory to explore the employee-organization relationship in the hospitality industry. Int. J. Contemp. Hosp. Manag. 33, 670–692. doi: 10.1108/IJCHM-06-2020-0538

[ref26] Meng B. Liu W. Zhou M. (2024). How can we boost learning and vitality? The influence of perceived organizational support on innovative behavior. Eurasian Bus. Rev. 14, 917–943. doi: 10.1007/s40821-024-00280-9

[ref27] Musenze I. A. Mayende T. S. Wampande A. J. Kasango J. Emojong O. R. (2021). Mechanism between perceived organizational support and work engagement: explanatory role of self-efficacy. J. Econ. Adm. Sci. 37, 471–495. doi: 10.1108/JEAS-02-2020-0016, 35579975

[ref28] Nazaretsky T. Ariely M. Cukurova M. Alexandron G. (2022). Teachers’ trust in AI-powered educational technology and a professional development program to improve it. Br. J. Educ. Technol. 53, 914–931. doi: 10.1111/bjet.13232

[ref29] Ochoa Pacheco P. Coello-Montecel D. (2023). Does psychological empowerment mediate the relationship between digital competencies and job performance? Comput. Hum. Behav. 140:107575. doi: 10.1016/j.chb.2022.107575

[ref30] Paterson T. A. Luthans F. Jeung W. (2014). Thriving at work: impact of psychological capital and supervisor support. J. Organ. Behav. 35, 434–446. doi: 10.1002/job.1907

[ref31] Poikkeus T. Suhonen R. Katajisto J. Leino-Kilpi H. (2020). Relationships between organizational and individual support, nurses' ethical competence, ethical safety, and work satisfaction. Health Care Manag. Rev. 45, 83–93. doi: 10.1097/HMR.0000000000000195, 29533273

[ref32] Porath C. Spreitzer G. Gibson C. Garnett F. G. (2012). Thriving at work: toward its measurement, construct validation, and theoretical refinement. J. Organ. Behav. 33, 250–275. doi: 10.1002/job.756

[ref33] Revelle W. (2026). psych: procedures for psychological, psychometric, and personality research. Available online at: https://cran.r-project.org/web/packages/psych/index.html (Accessed April 3, 2026).

[ref34] Rosseel Y. Jorgensen T. D. Wilde L. D. Oberski D. Byrnes J. Vanbrabant L. (2025). lavaan: latent variable analysis. Available online at: https://cran.r-project.org/web/packages/lavaan/index.html (Accessed April 3, 2026).

[ref35] Saad E. S. S. Elsayed S. M. (2019). Organizational support as perceived by staff nurses and its relation to their autonomy. EBNR 1:11. doi: 10.47104/ebnrojs3.v1i3.81

[ref36] Sitindaon M. Tj H. W. Tecualu M. (2021). The influence of organizational support and management of employee performance marketing in mediation by competence. Int. J. Sci. Technol. Manag. 2, 151–160. doi: 10.46729/ijstm.v2i1.102

[ref37] Spencer L. M. Spencer S. M. (1993). “Definition of a competency,” in Competence at Work: Models for Superior Performance, (New York: John Wiley & Sons, Ins), 9–11.

[ref38] Spreitzer G. M. (1995). Psychological empowerment in the workplace: dimensions, measurement and validation. Acad. Manag. J. 38, 1442–1465. doi: 10.2307/256865

[ref39] Spreitzer G. M. Porath C. (2014). “Self-determination as for thriving: building an integrative model of human growth at work,” in The Oxford Handbook of work Engagement, Motivation, and Self-Determination Theory, ed. Gagné M. (Oxford: Oxford University Press), 245–258.

[ref40] Spreitzer G. Sutcliffe K. Dutton J. Sonenshein S. Grant A. M. (2005). A socially embedded model of thriving at work. Organ. Sci. 16, 537–549. doi: 10.1287/orsc.1050.0153, 19642375

[ref41] Stinglhamber F. Caesens G. (2021). “Perceived organizational support,” in Essentials of Job Attitudes and other Workplace Psychological Constructs, eds. Sessa V. I. Bowling N. A. (New York: Routledge), 81–83.

[ref42] Tang D. Wen Z. (2020). Statistical approaches for testing common method bias: problems and suggestions. J. Psychol. Sci. 43, 215–223.

[ref43] Taylor R. A. Alfred M. V. (2010). Nurses' perceptions of the organizational supports needed for the delivery of culturally competent care. West. J. Nurs. Res. 32, 591–609. doi: 10.1177/0193945909354999, 20693336

[ref44] Thomas K. W. Velthouse B. A. (1990). Cognitive elements of empowerment: an "interpretive" model of intrinsic task motivation. Acad. Manag. Rev. 15, 666–681. doi: 10.5465/amr.1990.4310926

[ref45] Wang H. (2025). A Case Study on the Current State of Mental Health Education in Rural Junior High Schools — Based on a Survey of X Junior High School in Nanchang County. Nanchang: Jiangxi Normal University.

[ref46] Wang J. Liu Z. (2018). The effect of perceived organizational support on work-family conflict: the mediating role of teacher competence. Education Research Monthly, 65–70.

[ref47] Wang W. Liu X. (2019). Job characteristics and thriving at work of teachers: mediating of self-determination. Chin. J. Clin. Psychol. 27, 613–616.

[ref48] Wang W. Z. Wei Z. F. Wang Y. M. (2020). Effect of competence on professional development of physical education teachers in primary and secondary schools: the chain mediating role of professional identity and thriving at work. Chin. J. Clin. Psychol. 28, 1289–1292.

[ref49] Wang W. Wei Z. Wang Y. (2021). Job characteristics and thriving at work of physical education teachers in primary and secondary schools: the chain mediating role of perceived organizational support and competence. Chin. J. Clin. Psychol. 29, 1045–1049.

[ref50] Wang Z. Zhang D. J. (2011). Measurement of competencies of mental health teachers. J. Psychol. Sci. 34, 481–487.

[ref51] Wei H. Zhang P. (2025). Exploring the relationships between organizational support, teachers’ competency, emotion labor, and burnout in school-family collaboration. Teach. Teach. Educ. 165, 1–9. doi: 10.1016/j.tate.2025.105158

[ref52] Xu J. Fan Y. (2017). The evolution of the college English curriculum in China (1985-2015): changes, trends and conflicts. Lang. Policy 16, 267–289. doi: 10.1007/s10993-016-9407-1

[ref53] Xu L. Nilsson A. Zhu K. Engström M. (2025). Direct and indirect relationships between structural empowerment, professional competence, thriving at work and perceived stress symptoms: a cross-sectional correlational study on hospital nurses in a Chinese province. BMJ Open 15, e100696. doi: 10.1136/bmjopen-2025-100696, 41448701 PMC12742184

[ref54] Xu H. Wu C. Chen C. Yan B. Zha N. Zhang K. . (2025). The effect of illegitimate tasks on work withdrawal behavior of intensive care unit nurses: the chain mediating effect of perceived organizational support and thriving at work. Intensive Crit. Care Nurs. 90:104136. doi: 10.1016/j.iccn.2025.104136, 40578249

[ref55] Yang R. You X. Zhang Y. Lian L. Feng W. (2019). Teachers' mental health becoming worse: the case of China. Int. J. Educ. Dev. 70:102077. doi: 10.1016/j.ijedudev.2019.102077

[ref56] Yu X. Lin X. Xue D. Zhou H. (2024). Impact of work engagement on teachers' workplace well-being: a serial mediation model of perceived organizational support and psychological empowerment. SAGE Open 14, 1–14. doi: 10.1177/21582440241291344

[ref57] Zhuang C. (2019). Research on the Current Situation and the Relationship among Professional Competence, Job Satisfaction and Job Input for Psychological Teachers in Middle and Primary Schools. Fuzhou: Fujian Normal University.

[ref58] Zhuang J. Fan X. Yang N. Xiong Z. Chen Z. Pu J. . (2025). The effect of psychological empowerment on clinical nurses’ thriving at work: the mediating role of voice behavior. BMC Nurs. 24:1018. doi: 10.1186/s12912-025-03403-3, 40764956 PMC12323100

